# Oral Bait Immunization of Eurasian Wild Boar (*Sus scrofa*) Against African Swine Fever with “ASFV-G-ΔI177L”: Bait Performance, Immunogenicity, and Environmental Monitoring

**DOI:** 10.3390/vaccines14020193

**Published:** 2026-02-21

**Authors:** Jörg Beckmann, Sandra Blome, Nuria Bujan, Christian Gortázar, Theresa Holzum, Steffen Ortmann, David Relimpio, Alexander Schäfer, Elisenda Viaplana, Ad Vos, Virginia Friedrichs

**Affiliations:** 1 Nuremberg Zoo, 90480 Nuremberg, Germany; joerg.beckmann@stadt.nuernberg.de; 2 IUCN SSC Wild Pig Specialist Group, 50735 Cologne, Germany; 3 Tapir and Suiform Taxon Advisory Group of the European Association of Zoos and Aquaria, 1018 DC Amsterdam, The Netherlands; 4 Friedrich Loeffler Institut, 17493 Greifswald, Germany; theresa.holzum@fli.de (T.H.); alexander.schaefer@fli.de (A.S.); virginia.friedrichs@fli.de (V.F.); 5 Zoetis Manufacturing and Research, 17813 L’Hostalnou de Bianya (Olot), Spain; nuria.bujan@zoetis.com (N.B.); elisenda.viaplana@zoetis.com (E.V.); 6 SaBio, Instituto de Investigación en Recursos Cinegéticos (IREC, CSIC & UCLM), 13005 Ciudad Real, Spain; christian.gortazar@uclm.es (C.G.); david.relimpio@uclm.es (D.R.); 7 Ceva Tiergesundheit GmbH, 14469 Potsdam, Germany; steffen.ortmann@ceva.com; 8 Ceva Tiergesundheit GmbH, 33500 Libourne, France; ad.vos@ceva.com

**Keywords:** African swine fever virus, environmental DNA detection, immunogenicity, non-invasive sampling, oral vaccination, Suiformes, wild boar, conservation

## Abstract

**Background/Objectives**: African swine fever is currently the most devastating viral disease affecting domestic and wild suids, causing major economic losses and severe impacts on natural populations. Oral immunization could become an important tool to control the panzootic and support wild pig conservation. However, this requires safe and effective vaccines, baits accepted by target species, and vaccine reservoirs that reliably release the vaccine during bait intake while maintaining vaccine integrity. **Methods**: We evaluated different bait types and vaccine containers in four wild Suiformes species, including Eurasian wild boar. In the same wild boar, we assessed oral vaccination with the live attenuated vaccine candidate “ASFV-G-ΔI177L”. Environmental monitoring approaches were applied to detect potential virus shedding, and vaccine immunogenicity and dissemination were evaluated. Vaccine stability was tested in vitro in two container types under different temperature conditions. **Results**: Bait uptake and container performance varied between manufacturers and among species. Environmental samples were largely negative for vaccine virus genome under controlled laboratory conditions, with only a few positive cotton ropes (0.43% of all samples). After oral bait vaccination, 45% (9/20) of wild boar seroconverted, with a higher proportion in animals receiving the vaccine in the slightly less attractive bait (gelatine-based). Vaccine virus dissemination was limited to a small number of organs, including gastrohepatic and mandibular lymph nodes. **Conclusions**: Our findings demonstrate that wild pigs can be vaccinated orally with “ASFV-G-ΔI177L” while virus shedding appears minimal. Although the tested baits show potential for multiple target species, baits and containers require optimization. Environmental monitoring methods also need refinement for field application.

## 1. Introduction

African swine fever (ASF) is the most devastating viral disease affecting domestic and wild suids, causing immense global economic losses and severe impacts on natural populations and associated ecosystems. The causative agent, ASF virus (*Asfivirus haemorrhagiae* (ASFV)) [1], is a large double-stranded DNA virus of the family *Asfarviridae* [2]. Despite decades of intensive research, no treatment or globally licensed vaccine exist. In sub-Saharan Africa, ASFV circulates in a sylvatic cycle involving wild suids including the common warthog (*Phacochoerus africanus*) [2] and soft ticks of the genus *Ornithodoros*, which act as competent arthropod vectors. This ancient transmission cycle [3] reflects long-term coevolution between the virus and its hosts [4]. Common warthogs, as well as the other sub-Saharan African wild pig species desert warthogs (*Phacochoerus aethiopicus*), bushpigs (*Potamochoerus larvatus*) and red river hogs (*P. porcus*), but also giant forest hogs (*Hylochoerus meinertzhageni*), can become infected with ASFV, but typically show no clinical signs, indicating a high degree of tolerance to the disease [5,6,7,8]. In contrast, Eurasian wild boar (*Sus scrofa*) and domestic pigs (*S. s. domesticus*) develop severe, often hemorrhagic fever-like disease, with case fatality rates approaching 100% [9].

The global landscape of ASF changed dramatically in 2007 when ASFV genotype II was introduced from Africa into Europe (Georgia), likely through human-mediated activities [10]. This event marked the beginning of the ongoing global ASF panzootic [11]. Spillover from domestic pigs caused ASFV transmission into wild boar populations, leading to dramatic population declines, for example over 80% in Poland [12] and up to 87% in Malaysia [13]. Additionally, spillover events of ASFV from domestic to wild pigs are currently one of the biggest threats to wild pigs and one of the biggest challenges in wild pig conservation. While wild pigs are often perceived as agricultural pests because of crop damage [14], they are also keystone species and ecosystem engineers: they disperse seeds [15], enhance plant and insect diversity by rooting [16,17], are prey for large carnivores [18], and carcasses of wild pigs serve necrophages as a food source [19]. In many regions, wild pigs are also an important source of sustainable protein for local communities, linking ASF to broader One Health and food security concerns. Consequently, ASF’s ecological and socio-economic impacts extend far beyond livestock production. Especially the epidemic of ASF in Southeast Asia, more precisely Indonesia, the Philippines and India, poses a critical threat to wild pig diversity. This region harbors 12 of the world’s 17 wild pig species, and 11 of them are endemic to the region [20]. Since the first detections of ASFV in the named countries in 2019–2020, outbreaks have caused population declines of up to 90% in some areas [13,21]. Therefore, alternative control strategies compatible with conservation goals are urgently needed. Oral vaccination has proven to be a highly successful strategy and a game changer for regional eradication of viral diseases, e.g., classical swine fever (CSF) in wild boar [22]. Like in ASFV, traditional measures (hygiene, zoning, increased hunting) failed to eradicate CSF [23]. The success of such mass oral vaccination campaigns renders oral immunization against ASFV with vaccines in baits the most promising strategy for controlling ASF and the conservation of wild pigs. Especially endangered species would benefit, as culling or increased hunting are not viable options.

A prerequisite of successful oral vaccination is a widely licensed, effective, and safe vaccine, which should induce a population immunity of at least 40–50% for a robust interruption of disease spread. Live attenuated vaccines (LAVs) are the most promising approach in ASF vaccinology to date, demonstrating higher protection rates compared to, e.g., mRNA vaccines [24,25], inactivated vaccines [26], subunit vaccines [24,27], or vector vaccines [28]. For oral vaccination, a live vaccine is the most effective approach to date, and several LAV candidates have been evaluated for oral application in pigs: The naturally attenuated, non-hemadsorbing Latvian strain “Lv17/WB/Rie1” provided 83–92% protection in wild boar under experimental conditions [29,30], inducing strong antibody responses and preventing ASF-associated disease after challenge infection. Notably, vaccinated wild boar shed the vaccine virus, facilitating secondary transmission within populations. Similarly, the “ASFV-G-ΔMGF” vaccine candidate induced full protection in wild boar that responded to oral immunization [31]. Moreover, the vaccine candidate “ASFV-G-ΔI177L” has demonstrated full protection of pigs against lethal challenge following oro-nasal administration under controlled laboratory conditions [32].

Despite substantial progress, key challenges remain in achieving consistent immunogenicity, preserving vaccine stability, and ensuring reliable delivery via baits to wild pigs in nature. For effective oral vaccination, baits must be palatable, stable, and accepted across pig species and age classes, while minimizing uptake by non-target wildlife (e.g., birds). The bait matrix should remain functional under diverse climatic conditions and should be designed with sensory characteristics, i.e., scent, flavor, texture, color, and size. In addition, physico-chemical and operational properties, including thermal stability, water resistance, and mechanical durability (e.g., for aerial distribution) are important determinants of field performance. Incorporating uptake markers such as tetracycline has been widely applied in wildlife oral vaccination programs (e.g., rabies), but their implementation is associated with technical and regulatory constraints [33]. Alternatively, effective active and ideally non-invasive surveillance techniques like fecal sampling, swabs or environmental DNA could be advantageous to monitor the vaccination rates. The present study is embedded in the EU-funded project “African Swine Fever attenuated live Vaccines In Pigs” (ASFaVIP) and aimed to advance oral vaccination strategies against ASF for wild pigs by exploring the stability and immunogenicity of the “ASFV-G-∆I177L” vaccine upon dual oral bait application (response rate), as well as its specificity and acceptance among Eurasian wild boar of various ages. This candidate was selected because it is among the best-characterized ASFV LAVs and has demonstrated high immunogenicity and protection against lethal challenge following parenteral administration [32,34,35,36,37]. In addition, in a direct head-to-head comparison of oral vaccination candidates conducted within the ASFaVIP project, “ASFV-G-∆I177L” showed the most consistent oral performance and was therefore chosen as the lead candidate for further bait-based evaluation (Holzum et al., in preparation). At the same time, the use of modified-LAVs requires careful benefit–risk assessment [38]. Recent studies have reported safety concerns under specific experimental conditions, including adverse outcomes in pregnant animals and reversion-to-virulence phenomena following in vivo passages [39], underscoring the importance of rigorous safety evaluation and post-deployment monitoring. These aspects have been addressed in recent benefit–risk discussions and are considered essential when evaluating vaccination strategies against ASF [40]. First, baits were offered to two threatened Asian wild pig species: Sulawesi babirusa (*Babyrousa celebensis*) and Visayan warty pigs (*Sus cebifrons*), as well as Chacoan peccaries (*Catagonus wagneri*) in a zoo setting to identify the most attractive bait. Prior to the bait vaccination trial at the Friedrich-Loeffler-Institut (FLI), we executed a bait preference test and offered four different baits to Eurasian wild boar under laboratory conditions. Vaccine containers filled with dyed water were embedded into the baits to mimic the vaccine, to enable assessment of uptake and probability of vaccination. Afterwards, the animals were offered two types of vaccine-filled baits to assess the immunogenicity upon delivery. Additionally, we assessed the stability of the industry-grade vaccine virus “ASFV-G-ΔI177L” in respective vaccine containers upon storage at various temperatures over three months to evaluate environmental safety concerns and tested the feasibility of several non-invasive surveillance techniques for monitoring vaccination success.

## 2. Materials and Methods

The present study consists of two parts: part A describes a bait preference trial with Eurasian wild boar and zoo-housed Suiformes, where no vaccine was included. Part B describes the immunogenicity of orally administered “ASFV-G-∆I177L” in Eurasian wild boar only.

### 2.1. Animals

A total of 20 Eurasian wild boar of three age classes (juvenile < 12 months, sub-adult 12–24 months, adult > 24 months) and both sexes were used in the two-stage proof-of-concept study (testing of bait preference and immunogenicity) conducted under experimental conditions at FLI. To this end, animals were housed under biosafety level S2 conditions in indoor units with a floor area of 11 m^2^ (housing units 1–6) or 24 m^2^ (unit 7). Animals had ad libitum access to water and were fed commercial pig feed once a day. Positive reinforcement and enrichment items (e.g., apples, eggs) were used to facilitate gradual habituation to handling procedures. Due to biosafety requirements, organic bedding material could not be provided; however, rubber mats were installed to improve resting comfort. All pens were thoroughly cleaned daily. This animal trial received ethical approval from the regional competent authority (Landesamt für Landwirtschaft, Lebensmittelsicherheit und Fischerei Mecklenburg-Vorpommern [LALLF M-V]) under file reference 7221.3-1-023/25 (approved 2 July 2025) and was conducted in accordance with the German animal welfare law.

Upon arrival, the animals received an individual ear tag and were assigned to seven housing units. Two of the juvenile Eurasian wild boar were wild born but orphaned and consequently hand reared, all others were born under human care at FLI or in wildlife parks.

Due to differences in age, origin, and prior social familiarity, animals could not be randomized without risking severe dominance-related aggression. Therefore, housing units reflected social compatibility rather than experimental subgroups. For the immunogenicity study (part B), animals were assigned to two vaccination groups (*n* = 10 per bait type), while remaining in compatible stable units for welfare reasons. Thus, subdivision into stable units did not constitute independent experimental subgroups but was solely necessitated by animal welfare considerations. Serological outcomes were evaluated at the individual level within each vaccination group.

The Sulawesi babirusa, Visayan warty pigs, and Chacoan peccaries were all born in zoos. Details about all animals can be found in Table 1.

### 2.2. Study Part A: Bait Preference Trial

#### 2.2.1. Study Design

To select two baits for study part B (immunogenicity), four bait types (Table 2) were offered to all animals simultaneously in a heap (number of baits offered = number of individuals per pen × 2) on five consecutive days. This enabled selection of baits based on their attractiveness. To visualize oro-nasal contact with the subsequent vaccine, the different vaccine containers held water with blue food coloring as a surrogate for the vaccine. Bait attractiveness was assessed with a specific scoring system: no bait intake = 0 points, some baits bitten but containers not perforated/chewed = 1 point, most baits consumed and containers mostly perforated = 2 points, nearly all or all baits consumed and all or nearly all containers perforated = 3 points. Time to first intake, number of leftover baits and proportion of perforated/chewed vaccine containers were recorded. Depending on the groups/pens, assessment was done by either direct observation or by camera traps installed in the stables.

In addition, we offered the four bait matrices to Sulawesi babirusa, Visayan warty pigs and Chacoan peccaries housed at Nuremberg Zoo to test bait attractiveness and intake in threatened Suiformes. As baits tested in the zoo setting did not include vaccine containers, a modified scoring system was applied: no interest in the baits = 0 points, medium interest/intake = 1 point, high interest/intake = 2 points. In the zoo, the baits were offered only once to simulate naïve wild Suiformes that have not been in contact with the baits before.

#### 2.2.2. Baits and Vaccine Containers

We tested four different baits from two manufacturers in study part A (bait preference) to select one bait from each manufacturer for the study part B (immunogenicity) based on wild boar preference.

**Figure 1:**
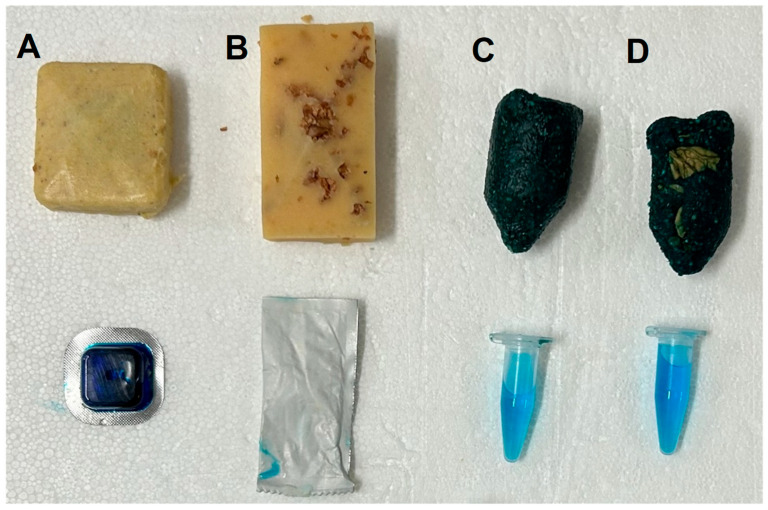
Overview of baits included in the bait-preference trial with Eurasian wild boar and zoo-housed Suiformes. Baits produced by Ceva (**A**,**B**) and IREC (**C**,**D**) were included in the trial. Corresponding vaccine containers are shown below the respective bait. Ceva baits included a plant fat-based (**A**) and a gelatine-based bait (**B**), while IREC baits were based on piglet feed and corn and offered as original formulation (**C**), as well as with crushed walnut shells as modification (**D**). Photograph: Beckmann, J. (2025).

Bait one (Ceva Pestiporc Oral (CPP)) was a commercial bait based on vegetable fat with an internal blister as the vaccine container, initially developed for oral vaccination of Eurasian wild boar against CSF. Bait two (Ceva Rabitec with modifications (CRT)), based on gelatine with an embedded sachet as the container, was initially developed for oral vaccination of carnivores against rabies [41]. The third bait (IBC) was produced by the Institute for Game and Wildlife Research (IREC) and is based on piglet feed and corn plus binders including honey and was designed for the oral vaccination of wild boar, with an Eppendorf tube inside as the vaccine container (patented with reference number ZP000453A) [42]. The fourth bait (IBW) was a modification of the IREC bait, with crushed walnut shells (*Juglans regia*) to increase attractiveness by scent, but also to induce micro lesions in the oral cavity during mastication to facilitate contact between vaccine virus and monocytes/macrophages. Properties of baits and vaccine containers are described in Table 2 and shown in Figure 1.

### 2.3. Study Part B: Immunogenicity Trial

#### 2.3.1. Study Design

For the vaccination and immunogenicity trial we used the modified Ceva Rabitec and IREC classic baits. For sachets, 1.5 mL of “ASFV-G-ΔI177L” with a formulated titer of 10^5.5^ HAD_50_/mL was filled in sachets, weld, and frozen at −80 °C before being embedded in the heated bait matrix (Ceva, Innovation Center, Potsdam, Germany). For the IREC baits, 1.5 mL was filled in 1.5 mL tubes (Eppendorf SafeLock #0030.120.086, Hamburg, Germany) and concealed in the bait matrix. The vaccine, a ready-to-use experimental vaccine with stabilizer, was manufactured to industrial standards on a proprietary permanent cell line (Zoetis Manufacturing and Research, Olot, Spain). The initial titer was confirmed by back-titration for IREC baits (10^5.5^ HAD_50_/mL), for Ceva baits back-titration revealed a titer of 10^4.5^ HAD_50_/mL. Depending on the acceptance of the two selected baits, sex, and age, the seven groups were divided into two subgroups (see Table 1): one was immunized by application of vaccine virus in the Ceva bait, the other with the IREC bait. When possible, each animal received one bait per immunization individually. The animals were immunized again 14 days after the initial vaccination (mimicking a double vaccination campaign). As application of “ASFV-G-ΔI177L” can cause significant clinical signs [39], we chose an adapted moderate humane endpoint (HEP) with 10 cumulative points [43]. At the end of the observation period 44/45 days post vaccination (dpv), the animals were subjected to necropsy.

#### 2.3.2. Sample Processing: Non-Invasive Sampling

**Figure 2:**
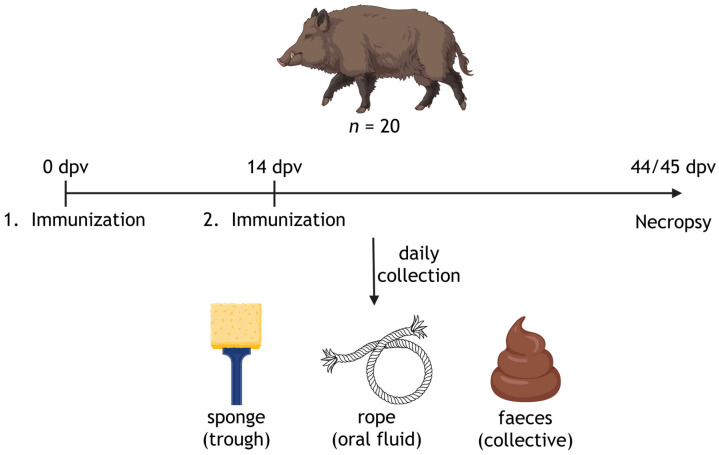
Graphical illustration of environmental sampling of wild boar pens following oral vaccination with “ASFV-G-∆I177L”. Collective samples containing oral fluids (trough swab, rope) and feces were collected daily during the study period. Created in BioRender.

To examine potential vaccine virus shedding following oral bait vaccination, multiple environmental samples were obtained once before and then daily after vaccination (Figure 2). This part of the study also aimed to evaluate the feasibility of collecting non-invasive samples from orally vaccinated wild boar. Cotton ropes (Farmshop, Stuttgart, Germany) were hung in each pen to collect oral fluids. A piece of chewed rope was taken each day and subjected to nucleic acid extraction. The feeding troughs were also swabbed daily using an Envirostik Kit (Technical Service Consultants Ltd., Heywood, UK). Fecal samples were collected every day by pooling portions from various fecal piles to obtain collective rather than individual samples. These were placed into dedicated feces collection tubes (Anders + Redelfs Laborbedarf, Weißenburg, Germany). Fluids from ropes, and Envirostick sponge-swabs were obtained by placing each rope or sponge into individual 50 mL centrifuge tubes on top of three closed 200 µL tubes, then centrifuged at 2000× *g* for 15 min at 15 °C. For nucleic acid extraction from pooled feces, 5 mL of 1 × PBS was added to each tube, vortexed to homogenize, and left at room temperature for 30 min. Subsequently, fecal samples were centrifuged at 2000× *g* for 10 min at 15 °C. Supernatants from the fecal suspensions and oral fluids were used for subsequent nucleic acid extraction.

#### 2.3.3. Sample Processing: Blood, Serum, and Organ Samples

Blood and serum were collected from the jugular vein (*Vena jugularis externa*, 0 dpv) or by cardiac puncture (at the end of the trial at 44/45 dpv) using aspiration tubes (KABE Labortechnik, Nümbrecht, Germany). Whole blood was analyzed by qPCR to determine viremia, while serum was subjected to ASFV-specific antibody detection assays.

Prior to sampling, all animals were sedated via darting (3 mL dart syringe with a 1.2 × 38 mm needle, Telinject, Dudenhofen, Germany) with a blow pipe (B31.C, Telinject, length 1 m, caliber 11 mm) and a mixture of 0.06 mg/kg medetomidine (Dormitor^®^, Vetquinol, Ismaning, Germany), 0.3 mg/kg midazolam (Ethypharm, Schönefeld, Germany), and 0.3 mg/kg butorphanol (Torbugesic^®^ vet, Zoetis, Berlin, Germany). After sample acquisition, anesthesia was reversed by intramuscular injection of 3.33 mg/kg atipamezole (Antisedan^®^, Vetquinol, Ismaning, Germany). As no animal reached the moderate HEP during the study, all necropsies were conducted at the end of the observation period. Animals were anesthetized as described and euthanized through intracardiac administration of pentobarbital sodium (Release^®^, WDT, Garbsen, Germany). Macroscopic lesions were recorded, and the following organs were collected to assess viral dissemination: tonsil, mandibular lymph node (mand LN), medial retropharyngeal lymph node (medret LN), salivary gland, lung, tracheobronchial lymph node (tra LN), spleen, kidney, gastrohepatic lymph node (gh LN), inguinal lymph node (ing LN), liver, and bone marrow. Urine was obtained individually by puncturing the bladder. All collected organs were subjected to nucleic acid extraction and qPCR to detect ASFV genome. Only PCR-positive samples (ct 35 or lower) were subjected to virus isolation to analyze presence of infectious particles. For tissue homogenates, about 200 mg of material was weighed into a 2 mL tube with 1 mL PBS and a 5 mm metal bead and homogenized for 3 min at 30 Hz in a TissueLyzer II (Qiagen, Hamburg, Germany).

#### 2.3.4. DNA Extraction and qPCR

Extraction of nucleic acids from 100 µL of EDTA blood, tissue homogenate, urine, or oral/fecal fluid was performed using the NucleoMag^®^ VET Kit (Macherey-Nagel, Düren, Germany) on a KingFisher™ Flex 96 platform (Thermo Fisher Scientific, Schwerte, Germany) as per manufacturer’s instructions. An ASFV genome-negative serum served as an extraction control. All qPCR reactions were performed using the virotype^®^ ASFV 2.0 PCR kit (Indical, Leipzig, Germany), including the manufacturer’s internal amplification control to monitor inhibition. To quantify ASFV DNA copy numbers in each sample, a standard with defined ASFV genome concentrations (10^0^–10^7^ copies/mL) was included in every run. Reactions were carried out on a Bio-Rad C1000™ thermal cycler equipped with a CFX96™ Real-Time System (BioRad, Grosskugel, Germany).

#### 2.3.5. Serology

All sera were tested for ASFV-specific antibodies by two commercial ELISA assays: ID Screen^®^ ASF Indirect (detection of ASFV-p32-targeting antibodies; ID.vet; Montpellier, France) and Ingezim PPA COMPAC (detection of ASFV-p72-targeting antibodies; Gold Standard Diagnostics; Dietzenbach, Germany). Procedures followed the manufacturers’ protocols. In parallel, all sera were analyzed via immunoperoxidase test (IPT), serving as the reference due to its higher sensitivity. Samples positive in IPT were also titrated in twofold dilutions starting from 1:40 to obtain semi-quantitative antibody titers.

#### 2.3.6. Hemadsorption Test and Virus Isolation

Hemadsorption tests (HATs) were used to determine the infectious titer of the experimental ASFV vaccine and to verify the presence of infectious ASFV in tissue samples. Since sachets containing 1.5 mL of the frozen vaccine suspension were submerged into bait matrix heated to 60 °C to prepare Ceva baits, a titer control was performed on once-thawed vaccine virus and vaccine virus recovered from a Ceva and an IREC bait. Peripheral blood mononuclear cells (PBMCs) were isolated from EDTA blood of a healthy donor pig housed in the FLI quarantine facility. The blood was diluted 1:10 with 10% Hanks dextran (Sigma-Aldrich, Darmstadt, Germany) and incubated for 90 min at room temperature. The PBMC-containing supernatant was washed twice with PBS, supplemented with 2.5 mM EDTA to prevent cell aggregation. The erythrocyte fraction was diluted 1:10 in PBS and stored at 4 °C. Isolated PBMCs were plated into 96-well Primaria™ plates (Corning, Darmstadt, Germany) at 5 × 10^5^ cells/well in DMEM supplemented with 10% FBS and 0.01% penicillin/streptomycin (Gibco, Paisley, Scotland). After 24 h incubation at 37 °C and 5% CO_2_, recombinant porcine CSF2 (2 ng/mL, KingFisher Biotech, Saint Paul, MN, USA) was added. Following an additional 24 h, the vaccine virus was titrated to confirm a titer of 10^6^ HAD_50_/mL. After 24 h post-infection, donor-specific erythrocytes were added at a ratio of 1:40 to initiate rosette formation and therefore detection of ASFV infected cells.

Virus isolation was conducted on qPCR-positive samples with ct values of 35 or lower. Isolated PBMCs were seeded in 24-well plates (2.5 × 10^6^ cells/well), differentiated with CSF2, and inoculated in duplicate with 100 µL tissue homogenate or collected fluid. After 72 h incubation, cells were frozen at −80 °C for cell lysis. Hemadsorption was then evaluated in quadruplicate replicates (*n* = 4) for each blind passage (*n* = 2). Results were categorized as negative (all wells negative) or positive (at least one well positive). Taking advantage of the mCherry reporter cassette encoded in the “ASFV-G-∆I177L” genome, HAT results were complemented by immunofluorescence analysis.

#### 2.3.7. Stability Assessment of Vaccine Virus

To assess the stability of the formulated vaccine virus under different ambient temperature conditions over time, industry-grade vaccine virus was combined with vaccine stabilizer and aliquoted into 1 mL portions, either in 1.5 mL SafeLock tubes (Eppendorf, Hamburg, Germany) or in sachets (Ceva, Innovation Center, Potsdam, Germany). To simulate field conditions of various regions and wild pig habitats, tubes and sachets were stored at 0 °C, 10 °C, 20 °C, 30 °C, and 40 °C. All samples were kept under these conditions for a total period of three months, with aliquots collected on incubation days 1 to 14 on a daily basis and then every 7 days until day 84. To account for experimental variation, each temperature and time point included replicate samples (*n* = 5 tubes and *n* = 5 sachets). The infectious titer of each sample was determined by HAT as described above.

## 3. Results

### 3.1. Study Part A: Bait Preference in Wild Boar and Other Wild Pig Species

Bait attractiveness to target species is crucial for successful oral vaccination campaigns, especially when several species in different habitats are involved. Therefore, we tested four different baits on four Suiformes.

The results of the pre-tests performed on Eurasian wild boar at FLI are shown in Table 3. Across all bait types, day 2 yielded the lowest score, whereas from day 3 to day 5 scores remained constant. The only exception was the CPP, which was less consumed on day 4 compared to days 3 and 5. Both IREC baits achieved higher scores (92 and 94) compared to the Ceva baits (75 and 78). In some cases, scoring of bait uptake was challenging, as nearly all baits were crushed/smeared/bitten into and spread. As we used the same containers in both IREC baits, assignment of individual containers to a specific IREC bait was not possible. Table 4 shows the results of the preference tests in zoo-housed Suiformes. In Visayan warty pigs, all four baits received 5 points. In Sulawesi babirusa, the CPP received 5 points, the two IREC baits 4 points each, and the CRT 2 points. In Chacoan peccaries, CPP received 2 points, both IREC baits 1 point each, the CRT bait 0 points. Among peccaries, only one adult female showed interest in the baits.

### 3.2. Stability Assessment of Vaccine Virus Infectivity “ASFV-G-∆I177L”

**Figure 3:**
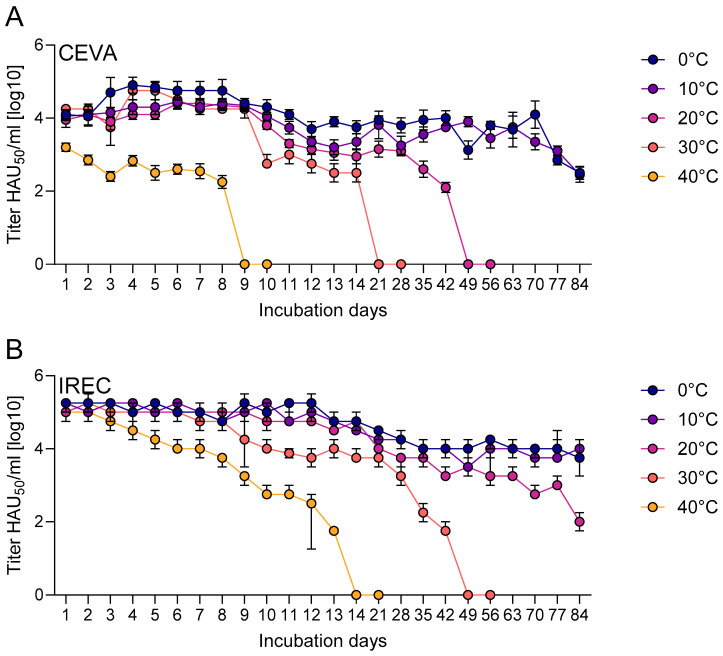
Stability assessment of “ASFV-G-∆I177L” in two different vaccine containers. A virus suspension with an initial titer of 10^5.25^ HAU_50_/mL (titrated at the start of the stability study) was either incubated in sachets (**A**) or tubes (**B**) at various ambient temperatures for up to 84 days. Points indicate the mean titer value of five replicates at each timepoint/temperature. Bars indicate the standard error of the mean. Infectivity was assessed by HAT; assessment was discontinued for the respective container/temperature after two consecutive negative results.

Titers of infectious vaccine virus were monitored over 84 days in two containers, sachets (Ceva) and Eppendorf tubes (IREC), under different incubation temperatures. In both systems, titers declined over time, with higher temperatures generally accelerating titer loss (Figure 3).

In sachets, the mean titer decreased across all temperatures from 10^5.25^ HAU_50_/mL (titrated at the beginning of the stability study) to 10^3.89^ HAU_50_/mL within the first 24 h (Figure 3A). In contrast, titers in tubes remained close to the initial value after 24 h, with a mean titer of 10^5.09^ HAU_50_/mL compared to an initial titer of 10^5.25^ HAU_50_/mL. In the sachets incubated at 40 °C, the titer dropped below the detection limit by day nine, while infectivity in the tubes dropped below the limit by day 14. At 30 °C, the titer in the sachets was below the detection limit by day 21, while infectivity persisted in the tubes until day 49. At 20 °C, the infectious virus could still be detected in the sachets until day 49, while the tubes maintained a detectable titer until the end of the observation period, with a titer of 10^1.95^ HAU_50_/mL at day 84. At 0 °C and 10 °C, the titer in the sachets declined to 10^2.5^ HAU_50_/mL and 10^2.45^ HAU_50_/mL at the end of the study on day 84, respectively. In the tubes, the titer dropped to 10^3.8^ HAU_50_/mL, and 10^3.95^ HAU_50_/mL by day 84, respectively.

### 3.3. Environmental Sampling

To evaluate the suitability of environmental sampling for detection of vaccine virus genome and ASFV-targeting antibodies, collective feces, trough swabs, and cotton ropes were collected daily from all pens throughout the study period. This is particularly relevant for field trials and applications, as we aimed to assess the feasibility of using oral fluids and fecal samples from wild boar to monitor shedding of vaccine virus and ASFV-specific antibody responses. If proven successful, such methods could serve as non-invasive strategies to evaluate vaccination success at baiting sites in the wild.

Detection of “ASFV-G-∆I177L” genome was unsuccessful in feces and trough swabs in the vast majority of samples (*n* = 929) throughout the study period (Supplementary Table S1), although 10^2^ genome copies per PCR run were reliably detected in pre-trial assessments (Supplementary Figure S1). In contrast, “ASFV-G-∆I177L” genome was detected in only four cotton ropes that had been chewed on by the animals. Specifically, 9.55 × 10^2^ and 9.5 × 10^2^ viral genome copies/mL of fluid were detected at 24 and 27 dpv in pen 7 (IREC), and 1.35 × 10^3^ and 2.2 × 10^3^ genome copies/mL were detected in pen 5 (Ceva) at 32 and 37 dpv, respectively (Supplementary Table S1). No ASFV-specific antibodies were detected in any environmental samples, as determined by IPT and commercially available competitive ELISA assays.

### 3.4. Clinical Observations

All wild boar remained healthy upon both immunizations, and no clinical signs were observed.

### 3.5. Assessment of Humoral Responses in Wild Boar After Dual Oral Vaccination

To evaluate the humoral immune response induced by oral vaccination, serum samples from all wild boar were analyzed for ASFV-specific antibodies. Serological testing was performed at the end of the animal trial to identify individuals that seroconverted. Successful seroconversion indicates induction of ASFV-specific adaptive immunity and provides insights into the immunogenic potential of “ASFV-G-∆I177L” when deployed in baits.

The initial assessment of seroconversion and the semi-quantitative titration of antibody titers was conducted by IPT (Table 5). In total, two animals in the IREC group (#77, #20) and seven animals in the Ceva group (#84, #85, #86, #87, #88, #80, #81) seroconverted. Importantly, seroconversion was also detected in one animal (#85) for which direct bait consumption had not been recorded. All other individuals remained negative in IPT.

**Figure 4:**
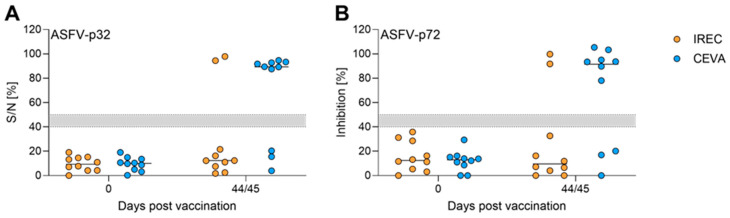
Humoral responses assessed by commercial ELISAs against (**A**) ASFV-p32 and (**B**) ASFV-p72 in wild boar (*n* = 20) after dual oral vaccination with 1.5 mL of 10^5.5^/mL (IREC) and 10^4.5^ HAU_50_/mL (Ceva) “ASFV-G-ΔI177L” via bait deployment. Animals that received the vaccine in an IREC bait are depicted as orange circles, animals that received the Ceva bait are depicted as blue circles. Gray areas between dotted lines indicate assay-specific thresholds, values above were considered positive.

Additionally, all sera were subjected to commercially available ASFV ELISAs (ID Screen African Swine Fever Competition, ID.vet, for detection of ASFV-p32-targeting antibodies (Figure 4A); and INgezim PPA Compac, Gold Standard Diagnostics, for ASFV-p72-targeting antibodies (Figure 4B)). All animals with positive IPT results were also clearly positive in the commercial ELISA.

### 3.6. Assessment of “ASFV-G-ΔI177L” Dissemination in Organs of Wild Boar

**Figure 5:**
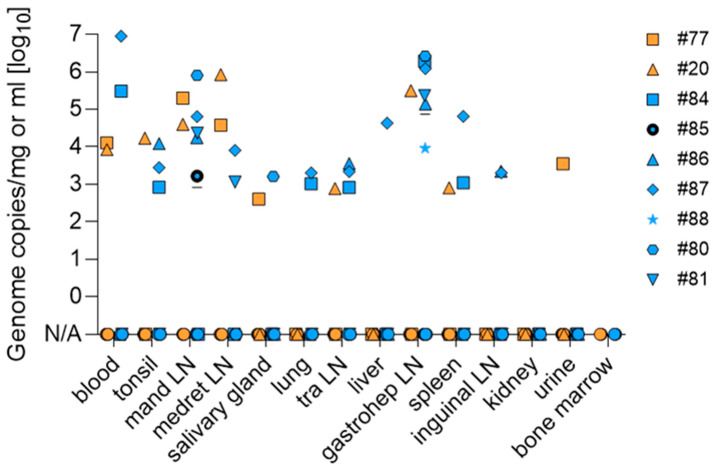
Detection of “ASFV-G-ΔI177L” genome copies in various tissues and urine of wild boar (*n* = 20) after dual oral vaccination. Samples were taken upon necropsies at 44/45 dpv. Animals received both vaccinations either in IREC (orange) or Ceva (blue) bait matrices. Individuals with at least one positive result in qPCR received a unique symbol, animals that remained fully negative are depicted as circles. N.A., no genome detected; mand LN = mandibular lymph node, medret LN = medial retropharyngeal lymph node, tra LN = tracheobronchial lymph node, gh LN = gastrohepatic lymph node; ing LN = inguinal lymph node.

Upon necropsy, various lymphoid tissues (gastrohepatic, mandibular, tracheobronchial, and medial retropharyngeal lymph nodes, spleen, bone marrow, and tonsil) and non-lymphoid tissues (salivary gland, lung, liver, and kidney), as well as urine samples, were collected. All samples were examined for the presence of “ASFV-G-ΔI177L” genome (Figure 5) and infectious particles (Table 6).

The two animals that received the IREC bait and developed an ASFV-specific humoral response (#77, #20) tested positive for viral genome in blood, tonsil, mand LN, tra LN, gh LN, and medret LN, salivary gland, spleen, and urine. Genome copy numbers ranged from 8.56 × 10^5^ copies/mg in medret LN of animal #20 to 4.06 × 10^2^ copies/mg in the salivary gland of animal #77. In contrast, the seven animals that seroconverted after consuming the Ceva baits (#84, #85, #86, #87, #88, #80, #81) further tested positive in lung and ing LN, with viral genome copy numbers ranging from 9.1 × 10^6^ copies/mL in the blood of animal #87 and 8.25 × 10^2^ copies/mg in the tra LN of animal #84. Overall, gh LNs yielded the highest viral genome copy numbers, with an average of 9.30 × 10^5^ genome copies/mg tissue. No viral genome was detected in the kidneys or bone marrow of any animal.

Complementary to the detection of viral genome in selected tissues, the presence of infectious ASFV particles was assessed in all samples with ct values of 35 or lower by HAT. The HAT was complemented by immunofluorescence analysis, as this method was shown to be more sensitive and utilized the mCherry reporter cassette encoded in “ASFV-G-ΔI177L”. Results are shown in Table 6. In animals vaccinated via consumption of the IREC bait, infectious ASFV particles were recovered from the mand LNs, gh LNs, and medret LNs. In animals that consumed the Ceva bait, infectious particles were detected in liver, mand LNs, and gh LNs. Overall, and in agreement with qPCR data, gh LNs yielded the most positive results, with 5/7 samples testing positive. No infectious viral particles were detected in tonsil, tra LNs, ing LNs or in spleen of any animal.

## 4. Discussion

In oral immunization of wildlife, baits are crucial as vehicles for delivering vaccines to target animals. Their design must account for species specific differences like diet and behavior, but also different age classes, especially when dealing with threatened wild pig species. In this study, four different baits were tested on Eurasian wild boar kept under laboratory conditions, as well as three species of zoo-kept wild Suiformes. This reflects the range of potential ASFV hosts.

Bait uptake by Eurasian wild boar increased between day 1 and 3, then stabilized, indicating accustomization and highlighting the value of pre-baiting to maximize vaccination campaign effectiveness. This is in line with field data, showing an increased uptake in pre-baited wild boar populations [44]. Piglet-feed-based IREC baits were overall preferred to Ceva baits, which can be explained by the integration of industrial pig feed in routine feed at FLI. Nevertheless, even common ingredients of pig feed like corn did not result in immediate acceptance, as observed with corn-containing CPP baits. This illustrates that omnivores can also have dietary preferences or are at least skeptical of unknown feed.

Zoo-housed Visayan warty pigs immediately accepted all bait types, whereas Sulawesi babirusa showed consistently low interest in gelatine-egg-based CRT, but good acceptance of other baits. Chacoan peccaries were overall reluctant to all bait types, with only one female mainly consuming CPP baits. These differences in preference match known species-specific dietary ecology: Visayan warty pigs and Sulawesi babirusa are known to be omnivorous, while Chacoan peccaries are predominantly herbivorous [20,45]. Chewing behavior and body size also affected bait uptake and container perforation. For example, Sulawesi babirusa divided larger IREC baits with their incisors and premolars, indicating that an embedded container would not have been ingested. Furthermore, juvenile Eurasian wild boar and Visayan warty pigs mostly broke IREC and CPP baits with their teeth on the ground, causing vaccine containers to be expelled or liquid to squirt on the floor rather than the mouth. Such feeding behaviors are reflected in crushed and damaged blisters found on the ground. It is likely that absorbent substrates would prevent adequate oral or even oro-nasal contact with the vaccine. Adult Eurasian wild boar mostly ingested whole baits and chewed the containers intensively, even swallowing them in some cases. This species- and size-dependency is consistent with general pig feeding behavior, where larger individuals can take up and grind larger particles, while smaller individuals must fragment more extensively. This is especially relevant, as juvenile individuals constitute a large proportion of wild pig populations and some species even remain small as adults (e.g., pygmy hogs (*Porcula salvania*), Visayan warty pigs). Therefore, bait formats should be adapted to smaller mouths and lower bite forces. This, however, might conflict with the necessary vaccine titers in small volumes. Furthermore, seasonal effects should be included in campaign design, as bait uptake in wild boar was shown to be higher in dry periods [46]. High ambient temperatures affect vaccine virus stability, as demonstrated in this study. However, the time window would be large enough (i.e., two weeks) for ambient temperatures of 20 °C and below.

In addition, the pigs used their forelegs to pin gelatine-based CRT baits to the ground, then tore them apart with their incisors. Sachets were often pulled out intact. Zoo-housed animals behaved similarly, even with gelatine-based Ceva baits that had been cut into halves. Our observations, together with the fact that pigs tend to sort out hard and inedible components, indicate that vaccine container and matrix should be strongly bonded. Additionally, containers should be soft and as small as possible to still contain sufficient vaccine volume but avoid recognition as foreign objects. It might be that straw-like vaccine containers coated with an attractive and familiar matrix (e.g., crushed corn) may aid thorough chewing and vaccine release into the oral cavity, which is essential for immune induction. Conclusively, these findings highlight that conservation-oriented vaccination campaigns against ASF should not rely on a single generic bait type across all Suiformes.

Stability of LAVs for oral wildlife vaccination remains a double-edged sword. Sufficient vaccine stability after bait deployment is essential so that enough target animals encounter a dose that is still effective [47]. However, excessive environmental stability is undesirable not only from an environmental safety perspective but also from a campaign management and surveillance perspective: vaccination campaigns should have a clearly definable endpoint, and prolonged persistence of biologically active bait material may cause biosafety issues or complicate interpretation of post-campaign monitoring data. It also may lead to repeated assessments when seropositive animals are detected after official campaign closure, an aspect of relevance in the context of notifiable animal diseases, where surveillance and control measures are adjusted based on the completion of vaccination activities.

The present study shows pronounced temperature and vaccine container dependent differences in “ASFV-G-ΔI177L” stability. Viral titers declined faster in sachets than in polypropylene tubes and the loss of infectivity was generally accelerated by higher temperatures. However, it must be kept in mind that the sachets were not from good manufacturing practice compliant fill-and-finish but rather prototype production. This means that the sachets were not strictly sterile and contained much more air than usual (meant for 3 mL liquid). In more detail, the initial titer in sachets dropped within the first 24 h from 10^5.25^ HAU_50_/mL to a mean of 10^3.89^ HAU_50_/mL, a level reached in the tubes on day 13 (10^3.99^ HAU_50_/mL). When incubated at 40 °C and 30 °C, the titer in sachets dropped below the detection limit at day nine and 21, respectively. In contrast, in tubes it persisted to days 14 and 49. At 0 °C and 10 °C, virus remained detectable in both until day 84, but with higher titers in tubes. These differences in degrading of the vaccine might relate to differences in material properties of internal surfaces and geometry of containers. The sachets had an approximately six times larger inner surface area compared to the tubes (approx. 4800 mm^2^ in the sachets vs. approx. 800 mm^2^ in the tubes), and therefore a larger potential reactive surface between virus and material. Guo et al. [48] reported that pores on surfaces of polyvinyl chloride (PVC) can act as virion traps, resulting in an eight times faster adsorption of virions compared to smooth surfaces of PVC. Changing temperatures, e.g., during handling, may enhance such effects via thermal expansion and contraction of the material. Additionally, hydrophobicity of the inner sachet layer, binding of serum proteins used as vaccine stabilizer, and leaching of additives with detergent-like properties have all been demonstrated to negatively impact virus/protein stability of polymer surfaces [49,50,51], which has also been demonstrated for ASFV [52].

Furthermore, differences in wall thickness (thin multilayer foil vs. 1.1 mm tube walls) possibly play a role only during short-term exposure to high temperatures, e.g., containers being sealed or submerged in heated bait matrix. However, the initial titer loss in sachets observed in both stability testing and back-titration of the vaccine used for vaccination of Eurasian wild boar, indicates that choice of container and bait manufacturing steps can immensely impact the dose in the bait. In comparison, tests with a vaccine candidate against CSF (“CP7 E2alf”) showed a lower temperature-dependent stability compared to “ASFV-G-∆I177L” but it was still successfully tested under field conditions. This highlights that the moderate degradation of the vaccine virus can be compatible with successful vaccination campaigns in the field [53].

In the context of ASF, the effects of temperature on the stability of vaccines must be considered not only for the oral vaccination of Eurasian wild boar in Mediterranean climates (e.g., Spain, Italy or North Africa), but also for the conservation of threatened wild pigs in tropical climates found in Indonesia or the Philippines. Therefore, local conditions (e.g., maximum temperatures in different seasons) should be considered when vaccination campaigns are planned. This might also require two or more vaccine deployments in short intervals. Nevertheless, further research on the impact of different container materials on the stability of vaccine viruses is needed to optimize the oral vaccination of wildlife.

An overall seroconversion rate of 45% after dual oral vaccination with “ASFV-G-ΔI177L” via baits is comparable to other seroconversion rates in similar studies using ASFV LAVs. However, in all other studies, the vaccine was applied to the tongue, avoiding bait-related losses. Barasona et al. [30] demonstrated a seroconversion rate in 66% of pigs orally immunized with “Lv17/WB/Rie1 ASFV”. Deutschmann et al. [31] reported 50% seroconversion in wild boar immunized with “ASFV-G-ΔMGF”. Under field conditions, achieving immunity of 40–50% in wild boar populations can effectively disrupt viral transmission chains as was shown for CSF oral vaccination of wild boar [54]. In our study, 45% of wild boar seroconverted, which holds important implications for the field especially considering the added complexity of bait uptake dynamics and container perforation. However, protection could only be proven by a lethal challenge experiment.

Despite the better acceptance of and higher titers in IREC baits compared to Ceva baits, 70% of animals seroconverted in the Ceva group compared to 20% in the IREC group. This discrepancy may be explained by characteristics of containers and bait matrices. The softer sachets in Ceva baits are less likely to be identified as foreign objects compared to the hard tubes in IREC baits, encouraging extensive chewing and enhancing contact between vaccine virus and mucosa/tonsils, which is a prerequisite for ASFV-specific immunity. Additionally, the gelatine-based Ceva matrix has limited capacity to absorb vaccine solution during chewing, whereas the piglet-feed-based IREC matrix that also contains honey, can rapidly absorb liquids. Honey itself has bioactive compounds, including antiviral ones [55]. Taken together, this ultimately may result in the vaccine being absorbed into the matrix and diverted into the gastrointestinal tract, without adequate exposure to the oral cavity. The original version of the IREC bait matrix in combination with tubes as containers was originally designed for the vaccination of wild boar against animal tuberculosis (TB) and tested first with recombinant *Escherichia coli*, where absorption of the vaccine into the matrix is advantageous [56]. Indeed, this system is feasible for TB immunization [57]. However, it might not be as well suited for ASFV immunization, highlighting that baiting systems are not easily transferable between pathogens. The difference in seroconversion rates between both baits, despite a lower titer in sachets (10^3.89^ HAU_50_/mL vs. 10^5.25^ HAU_50_/mL) suggests that the efficiency of vaccine delivery to the mucosa is significantly more critical than viral titer in the container. This dose-independency is further highlighted by the fact that wild boar #85 in pen 5 did not ingest any baits but seroconverted nevertheless after having contact with leftover baits of pen mates. This underlines that oral immunization is error-prone and is influenced by many factors, potentially including protease activity in saliva, microlesions or pH in the oral cavity. Both dose effects on vaccination efficiency and the strength of antibody responses warrant additional investigation. Tissue dissemination of both viral genome and infectious particles was consistent with previous studies using “ASFV-G-∆I177L”, in which vaccinated animals remained clinically healthy. The detection of viral genome in selected lymphoid tissues at necropsy reflects local replication following oral exposure rather than evidence of uncontrolled systemic spread. Importantly, genome detection does not equate to long-term persistence, high-level shedding, or transmission competence. To date, there is no evidence indicating the sustained long-term persistence of “ASFV-G-∆I177L” in clinically healthy wild boar under experimental conditions. Given the comparatively low number of re-isolations and viral genome copy numbers observed after oral vaccination and the inherent inefficiency of oral transmission at low doses, the likelihood of effective horizontal spread under these conditions appears limited. Nevertheless, the theoretical risk of prolonged persistence, transmission, or genetic change in modified-live vaccine strains warrants careful long-term evaluation.

Lastly, surveillance is key for evaluating the effectiveness of vaccination campaigns and to determine whether re-baiting is necessary. In principle, tools are available for both the direct detection of ASF (vaccine) virus and indirect detection of ASFV antibodies [58,59,60]. Although 45% of the animals seroconverted and a total of 929 environmental samples were tested, “ASFV-G-∆I177L” genome was found in only four rope samples, and no other sampling method yielded positive results. This corresponds to an overall detection rate of only 0.43%, and 1.41% in rope samples alone. From a biosafety perspective, this low detection rate indicates minimal environmental shedding under the conditions used. If such environmental sampling methods were applied in the field to evaluate the effectiveness and success of a vaccination campaign, our results would falsely suggest vaccination failure, as detection was limited to viral genome in periods of virus shedding, not antibody detection. However, our results indicate a potential for viral shedding in orally vaccinated Eurasian wild boar, as viral genome and/or infectious particles were found in the salivary glands and tonsils of some animals. Studies where pigs were inoculated with ASFV field strains, e.g., ASFV “Armenia07”, indicate that inoculation with field strains enables reliable detection of viral genome via environmental sampling, e.g., of feces, throughs, walls, and floors is possible [61,62,63]. Furthermore, several studies already described that for multiple attenuated ASFV strains, such as LAVs, viremia and virus shedding are more frequent. For example, in Eurasian wild boar vaccinated with the naturally attenuated ASFV “LV17/WB/Rie1”, viral genome can commonly be detected in blood and oro-nasal swabs; in animals vaccinated with “ASFV-G-ΔMGF360/505”, viral genome is detectable in blood and tissues, including salivary glands and mucosa [30,64]. Our limitations may be, in part, explained by the litterless environment and intensive daily cleaning.

Until now, “ASFV-G-ΔI177L” was generally regarded as a safe candidate, as associated safety studies reported low viraemia and hardly any shedding. Although more recent reversion to virulence studies report an increase in shedding [36], our results demonstrate that the conditions used in this study, which align with successful vaccination regimes, allow safe oral vaccination of Eurasian wild boar in the field. However, these results are reassuring from a biosafety perspective but showed that environmental sampling methods must be redefined in order to detect individuals that have been successfully immunized with “ASFV-G-∆I177L”.

## 5. Conclusions

Dual oral vaccination of Eurasian wild boar with “ASFV-G-ΔI177L” is feasible and can lead to 45% seroconversion rate, but is strongly influenced by bait design, vaccine stability, and host biology. A bait preference study including Eurasian wild boar and three threatened Suiformes revealed major species- and size-dependent differences in acceptance and chewing behavior. This indicates that matrices and containers must be adapted to species-specific diets and food processing behavior rather than relying on a single bait. Softer and smaller containers like sachets that adhere to the matrix seemed suitable for ensuring perforation and mucosa/tonsil exposure while remaining acceptable across taxa.

Stability experiments with “ASFV-G-ΔI177L” demonstrated temperature- and container-dependent degradation of vaccine virus, with faster titer loss in sachets than in polypropylene tubes. Additionally, this effect was accelerated at higher temperatures, e.g., 30 °C and 40 °C, while lower temperatures like 0 °C and 10 °C were less impactful. These data highlight the necessity to match container material and deployment schedules to local climatic conditions, especially in warmer regions. Furthermore, vaccine virus genome was only detected in 4 out of 929 environmental samples under controlled experimental conditions, hinting towards a favorable biosafety profile. However, non-invasive sampling methods need to be adjusted, as the current approaches were too insensitive to detect ASFV-specific antibodies or genome. In conclusion, our results provide robust guidance for designing ASF vaccination campaigns for wild Suiformes that are also conservation-compatible.

## Figures and Tables

**Table 1:** Species and characteristics of all animals included in this study. Housing units reflect social compatibility and animal welfare constraints. For the immunogenicity trial (part B), animals were allocated to two vaccination groups (Ceva bait, *n* = 10; IREC bait, *n* = 10) irrespective of pen structure.

**Wild Boar**				
**Housing Unit**	**Sex and Age**	**Origin**	**Rearing**	**Bait Used for Immunization**
1	1 adult female	FLI	parent	Ceva
2	1 adult male, 1 adult female	FLI	parent	IREC
3	1 juvenile male, 1 juvenile female	found orphaned	hand	IREC
4	3 juvenile females, 1 juvenile male	wildlife park	parent	IREC
5	3 juvenile females, 2 juvenile males	wildlife park	parent	Ceva
6	2 juvenile females, 2 juvenile males	wildlife park	parent	Ceva
7	1 subadult male, 1 adult female	wildlife park	parent	IREC
**Zoo-Housed Suiformes**				
**Group**	**Sex and Age**	**Origin**	**Rearing**	**Bait Used for Immunization**
Sulawesi babirusa	1 adult male, 2 adult females	zoo	parent	not immunized
Visayan warty pig	1 adult male, 2 adult females, 1 juvenile female	zoo	parent	not immunized
Chacoan peccary	1 adult male, 2 adult females, 3 juveniles	zoo	parent	not immunized

**Table 2:** Overview of properties of all baits and corresponding vaccine containers used in this study.

**Bait and Matrix Properties**				
Manufacturer	Ceva		IREC	
Type	Pestiporc oral (CPP)	Rabitec (CRT)	Classic (IBC)	Classic with crushed walnut shells (IBW)
Size	40 × 40 × 15 mm	65 × 35 × 20 mm	45–50 × 25 mm	45–50 × 25 mm
Weight	15 g	~25 g	~20 g	~20 g
Bait matrix	corn, fat	gelatine, egg powder, apple pomace	piglet feed, corn, sucrose, honey	piglet feed, corn, sucrose, honey, crushed walnut shells
Consistency	solid, crumbly	flexible, rubbery	solid, tough-elastic, crumbly	solid, tough-elastic, crumbly
Color	corn	corn	dark green	dark green
Melting point	30 °C	60 °C	none	none
**Vaccine Container Properties**				
Manufacturer	Ceva (sachet)		IREC (tube)	
Type	Pestiporc oral (CPP)	Rabitec (CRT)	Classic (IBC)	Classic with crushed walnut shells (IBW)
Size	30 × 30 × 7 mm	80 × 30 × 8 mm	41 × 10.7 mm	41 × 10.7 mm
Material	polyvinyl chloride (PVC) capsule sealed with aluminum foil	sachet with 3 laminated layers incl. aluminum foil	polypropylen (PP)	polypropylen (PP)
Consistency	rigid, inflexible (blister)	soft, flexible (sachet)	rigid, tough-elastic (tube)	rigid, tough-elastic (tube)

**Table 3:** Results of bait attractiveness and preference to Eurasian wild boar.

Manufacturer	Ceva		IREC		Daily Total Score
Type	Pestiporc Oral (CPP)	Rabitec (CRT)	Classic (IBC)	Classic with Crushed Walnut Shells (IBW)	
Day 1	17	13	17	19	66
Day 2	11	9	16	16	52
Day 3	18	18	20	20	76
Day 4	15	17	19	19	70
Day 5	17	18	20	20	75
Bait score	78	75	92	94	

**Table 4:** Results of bait preference testing in zoo-housed Suiformes.

Manufacturer	Ceva		IREC	
Type	Pestiporc Oral (CPP)	Rabitec (CRT)	Classic (IBC)	Classic with Crushed Walnut Shells (IBW)
Sulawesi babirusa male	2	0	1	1
Sulawesi babirusa female 1	2	1	2	2
Sulawesi babirusa female 2	2	1	1	1
Species score	6	2	4	4
Visayan warty pig male	2	1	1	1
Visayan warty pig female 1	1	2	2	2
Visayan warty pig female 2	2	2	2	2
Species score	5	5	5	5
Chacoan peccary *	2	0	1	1
Species score	2	0	1	1

* Only one adult female out of a group of seven animals showed interest in baits.

**Table 5:** ASFV-specific antibody titers detected in wild boar serum at 0 and 44/45 dpv via IPT.

Pen No.	Animal ID	0 dpv	44/45 dpv
1 (Ceva)	12	neg	neg
2 (IREC)	13	neg	neg
	14	neg	neg
3 (IREC)	99	neg	neg
	100	neg	neg
4 (IREC)	76	neg	neg
	77	neg	1/10,240
	78	neg	neg
	79	neg	neg
5 (Ceva)	84	neg	1/5120
	85	neg	1/5120
	86	neg	1/5120
	87	neg	1/5120
	88	neg	1/5120
6 (Ceva)	80	neg	1/10,240
	81	neg	1/5120
	172a	neg	neg
	172b	neg	neg
7 (IREC)	20	neg	1/20,480
	21	neg	neg

neg = negative.

**Table 6:** Detection of infectious ASFV particles in tissue samples of wild boar with ct values of 35 or lower.

	Organ							
Animal ID	Tonsil	Liver	tra LN	mand LN	gh LN	ing LN	Spleen	medret LN
77	/	/	/	pos	/	/	/	neg
84	/	/	/	/	pos	/	/	/
86	neg	/	neg	pos	pos	neg	/	/
87	/	pos	/	pos	pos	/	neg	neg
88	/	/	/	/	neg	/	/	/
80	/	/	/	pos	pos	/	/	/
81	/	/	/	neg	neg	/	/	/
20	neg	/	/	neg	pos	/	/	pos

/ = not tested, neg = negative, pos = positive. mand LN = mandibular lymph node, medret LN = medial retropharyngeal lymph node, tra LN = tracheobronchial lymph node, gh LN = gastrohepatic lymph node; ing LN = inguinal lymph node.

## Data Availability

All raw data generated in this study is available upon request to the corresponding author.

## Supplementary Materials

The following supporting information can be downloaded at: https://www.mdpi.com/article/10.3390/vaccines14020193/s1, Supplementary Report: Pre-trial assessment of ASFV vaccine virus genome detection from environmental sponge samples, including Supplementary Figure S1; Supplementary Table S1 (Viral genome copies per ml of fluid detected during non-invasive sampling of pens ((collective feces, trough swabs, cotton ropes).
