# Supplementary material for: Oral Bait Immunization of Eurasian Wild Boar (Sus scrofa) Against African Swine Fever with “ASFV-G-ΔI177L”: Bait Performance, Immunogenicity, and Environmental Monitoring
Source: Vaccines (Basel). 2026 Feb 21;14(2):193. doi: 10.3390/vaccines14020193 (PMC12945147; doi:10.3390/vaccines14020193)
Supplement: Supplementary file 1 [file vaccines-14-00193-s001.zip › Supplement1_Report_pre-trial_Envirostik.pdf]

Supplementary Report:

Pre-trial assessment of ASFV vaccine virus genome detection from environmental sponge samples

Trial design:

To evaluate the sensitivity of ASFV vaccine virus genome detection in environmental samples collected using cellulose sponges pre-moistened with 0.9% NaCl solution (EnviroStik, Technical Service Consultants Ltd.), a pre-trial assessment was performed as follows: A ten-fold dilution series was prepared from the experimental ASFV-G-ΔI177L vaccine (E22, batch 240506; titer 10^5 HAD_{50}/ml). For each dilution (undiluted and 10^{-1} to 10^{-5}), triplicate assays were conducted by spreading 100 µl of the virus suspension onto a metal carrier (60 × 15 mm) using a cover slip, followed by drying for 1 h at room temperature. The contaminated surfaces were then swabbed using cellulose sponges pre-moistened with 0.9% NaCl solution. Each sponge head was placed in a 50 ml centrifuge tube on top of three closed 200 µl tubes and centrifuged at 2,000 × g for 15 min at 15°C. The recovered suspension (~4 ml) was used directly for downstream analyses. Nucleic acids were extracted from 140 µl of suspension using the QIAamp Viral RNA Mini Kit (Qiagen) according to the manufacturer's instructions. Quantitative PCR was performed using the virotype® ASFV 2.0 PCR Kit (Indical), which includes an internal amplification control to monitor inhibition. For quantification of ASFV genome copies, a standard curve was included in each run, based on DNA standards containing 10^2–10^7 ASFV genome copies per ml. All qPCR reactions were run on a Bio-Rad C1000™ Thermal Cycler equipped with the CFX96™ Real-Time System (Bio-Rad). Data visualization was performed using GraphPad Prism 9 (GraphPad Software Inc.).

Results:

Viral genome was detected in all samples originating from metal carriers with undiluted vaccine (2.3 x 10^4 genome copies (gc) per PCR run) and from all 1:10 (1.5 x 10^3 gc per PCR run) and 1:100 dilutions (1.3 x 10^2 gc per PCR run). One positive reaction was recorded for the 1:1000 dilution (1.3 x 10^1 gc per PCR run). The results are depicted in Supplementary Figure S1.

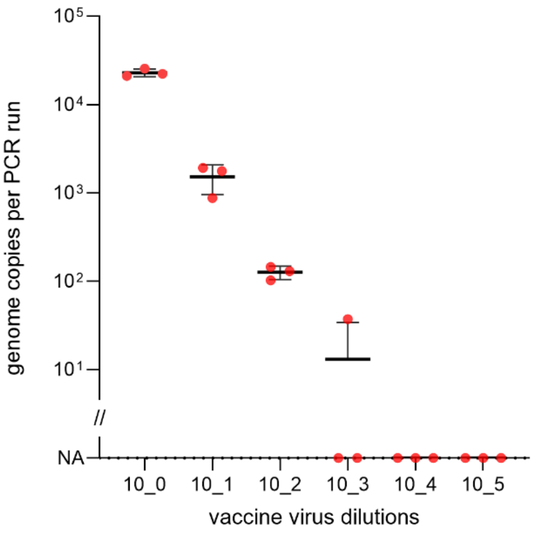

Supplementary Figure S1: Genome copy numbers per PCR run detected in samples from environmental sponge swab samples. Results are depicted as individual result and mean (error bars indicate standard deviation).
